# The Distribution of Nerves in the Dental Pulp and in the Dentine

**Published:** 1913-04-15

**Authors:** J. Howard Mummery


### THE DISTRIBUTION OF NERVES IN THE DENTAL PULP AND IN THE DENTINE.

BY J. HOWARD MUMMERY, M.R.C.S., L.D.S.

I do not think you need much persuasion to make you believe that the dentine is sensitive, and I do not think your patients will require any further conviction than your ministrations will afford. Still, John Hunter considered that dentine was not sensitive. Whether he argued on his own experience I do not know, because it was unlike John Hunter to draw conclusions from a single experience. Still, no one in his day appeared to have agreed with him that dentine was not sensitive. But we know that dentine is a very sensitive tissue indeed. Yet the problem of the mode in which the sensation is conveyed from it to the brain has long been an unsolved one. This is chiefly due to the difficulties connected with tracing organic elements in the hard substance of the tooth. The dentine having so long eluded observation, it was not much to be wondered at that the much more minute structure of the ultimate nerve fibrils escaped observation for so long a time, and it is only in consequence of the improved methods of histological research during the last few years that this investigation has been rendered possible. I will not dwell on the different views of innervation of the dentine, because I know Mr. Hopson has already dealt with that in his lectures. But I may say that for many years past it has been treated by most authorities as a doubtful question, some considering that the conduction of sensation through the dentinal fibril to the odontoblast cell would account for all the phenomena; others, such as Professor Kolliker, Professor Klein, and Mr. James Salter—all great authorities in dental histology—consider that the ultimate distribution of the nerves of the pulp has not been traced. And the two latter were of the opinion that it would ultimately be found that true nerve fibres ran in the dentinal tubes, and that the dentine would be brought into line in that way with the other tissues with regard to this mode of transmitting sensation. It seemed very unlikely that dentine should be an exception to all other tissues of the body and should not conduct sensation by means of nerve fibrils. The view held by Hopewell-Smith was that the odontoblast cells acted as a sort of ganglion cell through which sensation was transmitted. But there are many objections to this, one of them being that dentine is formed from the mesoblastic tissue, and the nerves from the epiblastic tissue; so mesoblastic odontoblast cells conduct from epiblastic tissue to nerve cells, which is always considered to be an impossibility.

With respect to my own investigations, I have long thought that true nerve fibres ended in dentinal tubes, and more than twenty years ago I had seen what I considered distinct evidence that such was the case. It is only in the last few years that I have been able to procure specimens which I considered adequately proved that the nerves did pass into the dentine. You are aware that the nerve fibres enter the pulp with the blood vessels at the apical foramen, that they enter in a considerable number of bundles of medullated fibres; in fact, the nerve supply of the pulp is so abundant that it is difficult to understand how it could be destined for the pulp alone. And another very striking point is that in longitudinal sections many of these nerve bundles traverse the pulp up to the crown at the cornu of the pulp without giving off any branches to speak of. There are some, but only a few.

But you see a large bundle of medullated fibres traverses the tooth to the cornu. I hope to show that these terminals sent off at this point and elsewhere in the pulp are not individual nerve fibres, but bundles of them; and the probability is that they are given off in the dentine. It is well known that the medullated fibres lose their sheath, or neurilemma, and their medullated sheath—which is probably their protective investment—during their passage through the tissues. And as they reach their destination these sheaths are lost, and they are distributed as neuro-fibrils or the elements of the axis cylinder of the nerve. This neuro-fibril is the essential part of the nerve fibre. It leaves the cell in the brain as a naked neuro-fibre; as it passes through the tissues it acquires a neurilemma, and further on still it sheds the neurillemma and again becomes a naked fibril. You will see it distributed to the dentine as a beaded fibril. I shall show you presently by means of slides what is evident under the microscope, and you can see sections on the table. The axis cylinder is shown made up of neuro-fibrils dotted or beaded. This is especially brought into view by chloride of gold stain, by the method I have been using lately, a modification of Lowits'. You will see the bundles of axis cylinders running alongside the blood-vessels, and the blood-vessels have taken on the contrast stain, namely, eosin. The medullated fibres subdivide in the pulp, and break up into very fine fibres distributed in the substances of the pulp. But most of these fine fibres are given off in the nerve fibres on the periphery of the pulp, and pass into a dense plexus immediately beneath the odontoblasts. This is called the plexus of Raschkow, after the histologist who described it. This plexus appears to be formed by an interlacement of fibres, rather than anastomosis; they seem intimately connected without being fused into one another. From this plexus fine fibres stretch out again in more or less parallel lines between and around the odontoblasts; they seem to involve the odontoblasts in a meshwork in many cases. Then they pass into a narrow plexus at the margin of the dentine. This plexus has been described as the terminal plexus of the nerves of the pulp. It was considered that this was the way in which the nerves of the pulp ended. But I have been able to show that from this marginal plexus as I have called it—we cannot now call it the terminal plexus—they again proceed and pass into the dentinal vessels. You will see that by the slides on the table; and they traverse the dentine in company with the dentinal fibril. They are very closely attached to the dentinal fibril, for often when the latter is pulled from the dentine you will see the little nerve fibres running alongside. This mode of distribution appears to occur at all parts of the periphery of the pulp, at the tip of the cornu. In two or three specimens which I have lately cut, the bundles of nerver fibres run up to the point I describe. They are serving some purpose in the pulp, and I have seen them sending off their fibrils and passing into the dentine in very much larger bundles than round *here*, where they go in very fine bundles. They are thicker bundles, but not thicker fibres. You will see the red-stained part is the bundle of medullated fibres. You cannot see the sheath, because it is not stained, except by a special osmic acid stain. There is a complete absence of the plexus of Raschkow here, but you will find it at other parts of the tooth. When I obtain the fresh material, which I am at present in want of, I shall endeavor to cut as many sections as possible in this plane to ascertain if this mode of termination at the cornu is constant. It is difficult to obtain a sufficient number of young teeth, although I have had the assistance of many friends. I must have young bicuspids, and if any of you should come across such, and will put them in 10 per cent. formalin for me, I shall be glad. They should, of course, be put in as soon after extraction as possible. In many preparations I find beaded fibres pass in close connection with the dentinal fibres, and more than one nerve for each tube. But it is difficult to tell how many go into a tube, because you never cut sections in which you have only one. And that makes dentine such a difficult subject to investigate because of the crossings and the undulations of the tubes. They can be traced as fine dotted lines into the dentine, and preparations have been successfully impregnated with nitrate of silver and with protargol, which is an organic salt of silver, showing the whole of the dentine permeated everywhere with delicate branches. It has been considered that these fine branches and tubes are very little visible in the crown of the tooth. There are very few under the enamel, and they are not otherwise very many of these fine branches. They are very plentiful in the root. These specimens show plainly that the whole of the dentine is permeated with these branches, and the reason we have not seen them is that we have not used the right stain to bring them out. The ordinary aniline stain will not do it. Nitrate of silver or protargol will show that the dentine is permeated with long delicate branches which show under the microscope and in the drawings. This is not an ordinary impregnation with nitrate of silver: they have to be impregnated with nitrate of silver and reduced with pyrogallic acid and formalin, and that brings down the nitrate of silver in black, and stains the dentine a deep brown. In the fine branches you can detect these dotted lines close up by the enamel, and close to the cementum even little dotted lines are clearly seen.

These lines are very fine indeed, and apparently represent the finer divisions of the neuro-fibrils which enter the pulp. It evidently shows that these dotted lines get finer and finer as they near the periphery of the tooth. The neuro-fibrils are capable of dividing. It is a point about which there had been some doubt, but there is now no doubt that the neuro-fibrils do divide in the tissues. The slides under the microscopes show these divisions and lines with very great distinctness. The same is seen at the junction of enamel and dentine, where the dentinal tubes are coarser than at the cement margin; many of the tubes look truncated, as if they had been cut off. That is explained by the dentine undergoing, during development, an interchange of absorption and deposition. It is little understood why that should occur, but it looks as if it is so. These truncated ends of the tubes you do not see under the cementum. But at all events you can trace in the fine tubes under the enamel the same thing. I therefore consider that the true terminations of the nerves are by fine arborizations of these neuro-fibrils beneath the enamel and cementum. In regard to where the dentinal tubes penetrate the enamel, and in the so-called spindles which you often see in teeth, the curious projections into the enamel, I cannot say whether the nerve fibres penetrate into these, because it is exceedingly difficult to get a thin section of enamel and dentine in contact by the Weil process, which is the only one to show it. If I can get a tooth and happen to cut it in the right plane to get these spindles, we can see whether these dotted fibres pass in. Professor Reaumer says they do, and that this is the final termination of the nerve fibres in the pulp.

He said he saw nerve end bodies stained red by chloride of gold. I now show you the section of a prehistoric tooth taken from one of the British battlefields. I am sure, from the appearance of the spindles in this case, you would say there was a nerve end body in the spindle. That was a tooth which had been dried for centuries. That looks so much like it that it is easy to understand how one might be mistaken in looking at these highly refractive bodies. It is possible that nerve endings are there, but I do not think it has been proved. The specimen which I spoke of makes one very careful about giving an opinion.

It seems astonishing that such an enormous number of nerves should be distributed to the dentine. It is difficult to understand, and it has been suggested that these nerves are not all nerves of sensation, but that many of them may be trophic nerves. Of course, all cells which are engaged in active secretion have nerves, and it is possible that this has some connection with calcification of the tooth. There is no doubt that in the pulp you sometimes see non-medullated nerves. I have seen many, especially by the Weil method. You can often see non-medullated fibres accompanying the medullated fibres.—*British Dental Journal.*

